# Physical activity and academic achievement across the curriculum (A + PAAC): rationale and design of a 3-year, cluster-randomized trial

**DOI:** 10.1186/1471-2458-13-307

**Published:** 2013-04-08

**Authors:** Joseph E Donnelly, Jerry L Greene, Cheryl A Gibson, Debra K Sullivan, David M Hansen, Charles H Hillman, John Poggio, Matthew S Mayo, Bryan K Smith, Kate Lambourne, Stephen D Herrmann, Mark Scudder, Jessica L Betts, Jeffery J Honas, Richard A Washburn

**Affiliations:** 1 Cardiovascular Research Institute, Division of Internal Medicine, The University of Kansas Medical Center, 3901 Rainbow Boulevard, Kansas City, KS, 66160, USA; 2 Department of Health, Sport, and Exercise Science, The University of Kansas, 1301 West Campus Road, Lawrence, KS, 66045, USA; 3 Department of Internal Medicine, The University of Kansas Medical Center, 3901 Rainbow Boulevard, Kansas City, KS, 66160, USA; 4 Department of Dietetics and Nutrition, The University of Kansas Medical Center, 3901 Rainbow Boulevard, Kansas City, KS, 66160, USA; 5 Department of Psychology and Research in Education, The University of Kansas, 1122 West Campus Road, Lawrence, KS, 66045, USA; 6 Department of Kinesiology and Community Health, The University of Illinois Urbana-Champaign, 906 South Goodwin, Urbana, Il, 61801, USA; 7 Department of Biostatistics, The University of Kansas Medical Center, 3901 Rainbow Boulevard, Kansas City, KS, 66160, USA; 8 Department of Kinesiology and Health Education, Southern Illinois University Edwardsville, Vadalabene Center, Box 1126, Edwardsville, IL, 62026, USA; 9 The University of Kansas-Lawrence, Robinson Center, Rm. 100, 1301 Sunnyside Avenue, Lawrence, KS, 66045, USA

**Keywords:** Physical activity, Children, Academic achievement, Cluster-randomized trial, Cognitive function, Fitness, Attention to task

## Abstract

**Background:**

Improving academic achievement and reducing the rates of obesity in elementary school students are both of considerable interest. Increased physical activity during academic instruction time during school offers a potential intervention to address both issues. A program titled “Physical Activity Across the Curriculum” (PAAC) was developed in which classroom teachers in 22 elementary schools were trained to deliver academic instruction using physical activity with a primary aim of preventing increased BMI. A secondary analysis of data assessed the impact of PAAC on academic achievement using the Weschler Individual Achievement Test-II and significant improvements were shown for reading, math and spelling in students who participated in PAAC. Based on the results from PAAC, an adequately powered trial will be conducted to assess differences in academic achievement between intervention and control schools called, “Academic Achievement and Physical Activity Across the Curriculum (A + PAAC).”

**Methods/design:**

Seventeen elementary schools were cluster randomized to A + PAAC or control for a 3-year trial. Classroom teachers were trained to deliver academic instruction through moderate-to-vigorous physical activity with a target of 100+ minutes of A + PAAC activities per week. The primary outcome measure is academic achievement measured by the Weschler Individual Achievement Test-III, which was administered at baseline (Fall 2011) and will be repeated in the spring of each year by assessors blinded to condition. Potential mediators of any association between A + PAAC and academic achievement will be examined on the same schedule and include changes in cognitive function, cardiovascular fitness, daily physical activity, BMI, and attention-to-task. An extensive process analysis will be conducted to document the fidelity of the intervention. School and student recruitment/randomization, teacher training, and baseline testing for A + PAAC have been completed. Nine schools were randomized to the intervention and 8 to control. A random sample of students in each school, stratified by gender and grade (A + PAAC = 370, Control = 317), was selected for outcome assessments from those who provided parental consent/child assent. Baseline data by intervention group are presented.

**Discussion:**

If successful, the A + PAAC approach could be easily and inexpensively scaled and disseminated across elementary schools to improve both educational quality and health. Funding source: R01- DK85317. Trial registration: US NIH Clinical Trials, http://NCT01699295.

## Background

Improving academic achievement and reducing obesity and its associated negative health consequences in elementary school students are important goals. School based interventions that provide increased physical activity, primarily through physical education (PE), have the potential to address both issues; however, concern over meeting goals for academic achievement has resulted in the reduction or elimination of PE in many elementary schools. This is unfortunate as evidence is accumulating to suggest that obesity has a negative impact on academic achievement, while increasing physical activity may both reduce the level of obesity and improve academic achievement. Cross-sectional studies on the associations between physical activity, primarily during PE, and/or cardiovascular fitness and academic achievement, are generally positive [1,2]. However, results from the few available prospective studies on this topic are inconsistent [3-6]. The disparate results are likely a function of differences in study design, measures of academic achievement (i.e., class grades, non-standardized or standardized achievement tests), dose and type of physical activity intervention (i.e., enhanced PE with an emphasis on muscular or cardiovascular fitness, balance/coordination, classroom physical activity breaks), sample characteristics (i.e., size, age, gender, race/ethnicity), and the duration of the intervention (7 weeks - 6 years). PE in elementary schools is generally restricted to 30-minute sessions, 2 or 3 days/week, and provides limited amounts of moderate-to-vigorous activity (MVPA) due to large class sizes, lack of space and equipment, and in some cases, inadequate instructor training [7-9]. Therefore, PE may not be the ideal venue for increasing physical activity in the elementary school setting.

To provide increased MVPA while maintaining academic instruction time, a program titled “Physical Activity Across the Curriculum” (PAAC) was developed in which elementary school classroom teachers were trained to deliver academic lessons using MVPA with a primary aim to prevent increased BMI [10,11]. The impact of PAAC (2–10 minute lessons/day, 5 days/week) on academic achievement was assessed as a secondary analysis in a 3-year cluster randomized trial (PAAC vs. control) at baseline and at the end of year 3 in a random sub-sample of students (n = 252), in grades 2 or 3 at baseline, using the Weschler Individual Achievement Test-Second Edition (WIAT-II) [12]. Significant improvements were shown for all academic measures in students attending PAAC, but not for students attending control schools. Based on the evidence from both the literature [1,2,6,13-16] and the PAAC study [10,11], a 3-year trial will be conducted to evaluate the impact of physically active instruction on academic achievement in elementary school students in grades 2 and 3 at baseline. A + PAAC will alter the classroom environment by providing existing academic lessons taught using physical activity. Thus, the amount of time devoted to academic instruction is not diminished. The primary aim of this study is to assess differences in academic achievement in students who receive physically active lessons and students in control schools who receive regular academic lessons. Secondary aims include determining potential mediators of any association between A + PAAC and academic achievement, including changes in changes in cognitive function, cardiovascular fitness, daily physical activity, BMI and attention-to-task. An extensive process analysis will also be performed to document the fidelity of the intervention. This article describes the rationale and design of this study and presents baseline characteristics of both the participating schools and the study sample. Other data collected at baseline is currently being analyzed and will be the focus of forthcoming manuscripts.

## Methods

### Study design and setting

Seventeen elementary schools in northeast Kansas were stratified by school district and cluster randomized to receive A + PAAC (N = 9) or serve as non-intervention controls (N = 8). Schools will be monetarily compensated for participation; 50% following randomization and 50% at study completion. All classroom teachers in A + PAAC schools were trained to deliver two 10-minute physically active academic lessons 5 days/week over the 3-year intervention. Teachers in control schools were asked continue to use traditional classroom instruction and students in both intervention and control schools will continue with their typical PE schedule (2–30 minute classes/week). A random sample of 2^nd^ and 3^rd^ grade students in each school was selected from those who provided parental consent/child assent (stratified by grade and gender) to complete outcome assessments including academic achievement, cognitive function, cardiovascular fitness, daily physical activity, and BMI at baseline (Fall 2011) and at the end of the spring semester for the next 3 years (2012–2014). Only students in 2^nd^ and 3^rd^ grade at baseline were assessed, as they will remain in elementary school for the duration of the 3-year intervention. School and student recruitment school randomization, teacher training, and baseline testing for A + PAAC have been completed. This study was approved by the Human Subjects Committee at the University of Kansas (HSCL #13460).

### Recruitment

The parents of students in grades 2 and 3 in both the A + PAAC and control schools received a brief flyer describing the study, including exclusion criteria and assessment procedures. Parents of students interested in participation returned a portion of the flyer providing their contact information to the school. The parents were then invited to attend an informal meeting at the school with both study staff and classroom teachers where the study was described. Descriptive characteristics of both the intervention and control schools are presented in Table 1.

**Table 1:** Descriptive characteristics of schools participating in the study

	**Intervention**	**Control**
Total School Enrollment	369.7 ± 96.6	385 ± 121.9
Total 2^nd^ and 3^rd^ Grade Enrollment (N = 1902)	978	924
2^nd^ and 3^rd^ Grade Class Size	22.5 ± 5.3	20.4 ± 2.6
Urban/Rural Schools (n/n)	6/3	6/2
Minority (%)	23.8	30.2
Free/reduced lunches (%)	35.3	42.5

Values are N’s or mean ± standard deviation.

Students with physical or intellectual disabilities or learning disorders will receive A + PAAC or control as a function of attending a participating school; however, some students were ineligible to complete outcome assessments due to their disability (i.e. blind, severe intellectual disability, etc.). Baseline participant characteristics by intervention group are presented in Table 2.

**Table 2:** A + PAAC: participant baseline characteristics

	**Intervention** **(n = 370)**	**Control** **(n = 317)**
Age (years)	7.6 ± 0.6	7.6 ± 0.6
Height (cm)	129.6 ± 6.5	129.6 ± 6.9
Weight (kg)	29.6 ± 7.2	29.6 ± 7.7
Waist (cm)	57.6 ± 7.5	57.1 ± 7.7
BMI Percentile	61.1 ± 29.3	61.1 ± 30.0
Female (%)	47.9	53.1
Minority (%)	16.3	23.2
Free/reduced lunches (%)	26.5	36.7

Values are mean ± standard deviation unless otherwise indicated.

*Note*. 698 students were randomized. 687 performed anthropometric testing.

### A + PAAC lessons: description

A + PAAC lessons can be used in a variety of academic disciplines including math, language arts, geography, history, spelling, science, and health. For example, students might hop or skip across the classroom and count their own “laps” as well as add laps of groups of students (i.e., 5 students X 5 laps each = 25). Geography (north, south, east, west) can be taught by having students run to the appropriate area designated for one of the directions. For example, if Texas is called, students would run or skip to the south side of the classroom. A + PAAC simply represents a “concept” whereby physical activity is integrated with classroom academic instruction. Thus, the scope of physically active lessons is limited only by the creativity of the teacher.

### A + PAAC: prescribed amount/intensity

Teachers will be asked to deliver two, 10-minute A + PAAC lessons per day, one lesson in the morning and one in the afternoon, 5 days per week. This will result in a weekly total of school based physical activity of ~160 minute/week (A + PAAC = 100 minutes; PE = 60 minutes) which is slightly greater than the minimum 150 minutes/week of physical activity for children as recommended by Healthy People 2010 [17]. A + PAAC lessons will provide MVPA with an energy expenditure of 4–5 METs [18] that will be verified by indirect calorimetry. Some recommendations call for 60 minutes/day of MVPA for health promotion in children [19]. However, based on focus group feedback from our pilot study [20], it is unlikely that school administrators and teachers would tolerate this level of physical activity in the classroom.

### A + PAAC: classroom teacher training

Teachers in intervention schools were trained to deliver A + PAAC during a 6-hour in-service session conducted at the school prior to implementation of the intervention. Initial teacher training will be followed by individual meetings with study staff throughout the intervention to provide one-on-one feedback regarding the quality of intervention implementation. The use of an initial workshop is consistent with existing teacher training in-service schedules utilized in the participating school districts and is also similar to the training scheduled employed in other school based physical activity interventions including Sports, Play, and Active Recreation for Kids (SPARK) [21] and Child and Adolescent Trial for Cardiovascular Health (CATCH) [22]. All training sessions were conducted by a member of the research staff who has over 25 years of experience working with elementary schools to increase physical activity in children. Teachers were asked to personally engage in the A + PAAC lessons to model the active behavior for the students. During training, the research team and teachers had the opportunity to share ideas regarding the development of new A + PAAC activities and to devise innovative approaches for the delivery of the A + PAAC program. On completion of training, A + PAAC teachers have the skills and knowledge to reduce lesson planning time, make smooth transitions to incorporate physical activity into the curriculum, and be active role models for increasing classroom physical activity in their students. To assist teachers in delivering A + PAAC lessons, a written teacher’s guide and access to the study website was provided, which includes a variety of sample lessons with associated energy expenditure values (METs). A 1-day (4-hour) in-service will be conducted at the beginning of intervention years 2 and 3 to provide feedback to teachers regarding the intervention from the previous year and to discuss implementation of the A + PAAC intervention.

### Outcome measures

#### Research staff training and blinding

All outcome measures were assessed at baseline (Fall 2011) and will be repeated each Spring over the 3 year intervention. Two groups of research staff were trained by members of the investigative team to obtain outcome and process assessments. One group was trained on the observations of physical activity in the classroom and attention-to-task, while the second group was trained on the assessment of academic achievement, cognitive function, daily physical activity, height/weight, and cardiovascular fitness. Prior to data collection and then at the beginning of each data collection period, research staff will participate in several training sessions to review and practice standardized protocols for all assessments. Inter-rater reliability of > 0.90 will be required of all research staff prior to participation in data collection. Research staff obtaining measures of physical activity and attention-to-task in the classroom cannot be blinded to intervention group. However, staff obtaining all other assessments and those performing data entry will be blinded to intervention group.

### Academic achievement

Academic achievement was assessed using the Weschsler Individual Achievement Test-Third Edition (WIAT-III) [23] and the Kansas Assessment, a standardized academic achievement test administered to 3^rd^ through 12^th^ grade students annually by the state of Kansas. The WIAT-III was individually administered by research staff trained and supervised by a qualified co-investigator. Five WIAT-III subtests (reading comprehension and oral reading fluency, spelling, and mathematics problem solving and numerical operations) were selected and take approximately 40–50 minutes to complete. The WIAT-III has excellent inter-scorer reliability (i.e., 0.92 to 0.99), internal consistency, split-half method (e.g., by age-range from 0.83 to 0.98), and test-retest stability (e.g., for children 6 to 12 years of age, 0.87 to 0.96 over 2 to 32 days). Validity is supported via item reviews of curriculum experts and by correlations with other tests, including the WIAT-II (e.g., .62 to .86), and measures of academic achievement (e.g., 0.60 to 0.82) [23]. The WIAT-III was scored by the research staff and all tests were checked for accuracy by a trained investigator. Any discrepancy in scores between examiner and the research team member was resolved by a co-investigator. All scores were entered into the WIAT-III computerized scoring assistant, which automatically disallows out-of-range values and computes subtest and composite scores. In addition to WIAT-III, the individual results of the Kansas State Assessment (KSA) standardized achievement tests will be obtained. These tests evaluate performance on the Kansas State standards, which define what students should be learning each year and are administered annually to children in grades 3 and higher. Two years of results for children initially in 2^nd^ grade and 3 years of results for children initially in 3^rd^ grade will be obtained.

#### Cognitive function: modified Eriksen flanker task

Cognitive control was evaluated using a computer based modified Eriksen flanker task [24]. Since pre-adolescent children were tested, the task will employ cartoon fish facing different directions rather than letters to reduce the working memory requirements [16,25]. A fish facing to the right “>” will require a right-handed response and a fish facing to the left “<” will require a left-handed response. These two response-eliciting stimuli were flanked by an array of other cartoon fish that are congruent (>>>> > or < <<<<), or incongruent (> > <> > or < <> < <) to the centrally placed target stimulus. The incongruent condition requires greater amounts of cognitive control (i.e., inhibition), which results in a response delay due to activation of the incorrect response (elicited by the flanker stimuli) before evaluation is completed, and this response competes with the correct response that is elicited by the central stimulus [26]. Congruent and incongruent conditions were equi-probable (0.5). Upon completion, the response requirements changed on the next task such that the participants responded in the opposite manner (e.g., right-handed response for a left-facing fish and vice-versa). Participants completed two blocks consisting of 100 trials each. The outcome measures are reaction time and response accuracy. This test (including rest between blocks) can be completed in less than 15 minutes.

#### Cognitive function: spatial n-back task

The purpose of the n-back task is to provide a continuous load on working memory, requiring updating and reorganization of memory representations. A modified version of the spatial n-back task includes six white-framed boxes, each measuring 4 × 4 cm, spaced in a circular fashion 9.5 cm from a centrally-presented fixation cross. For each trial, an illustrated black and white cow appeared pseudo-randomly inside one of the six boxes. For the 0-back condition, participants were instructed to respond as quickly and accurately as possible with a right button press when the cow appears in the upper-right box (i.e., target), and with a left button press when the cow appears in any of the remaining five boxes (i.e., correct reject). For the 1-back and 2-back conditions, participants were instructed to respond as quickly and accurately as possible with a right button press if the cow appears in the same box as the previous trial (i.e., target) for the 1-back condition, and two trials previous for the 2-back condition. A left button press is indicated if the cow is in any of the four remaining locations (i.e., correct reject). Targets were presented with 33.3% probability, with the 0-back condition containing 45 trials with 15 targets, and the 1- and 2-back containing 72 trials with 24 targets each. The outcome measures are reaction time and response accuracy. This test (including rest between blocks) can be completed in approximately 15 minutes.

### Anthropometrics (Height/weight/waist circumference)

Children were weighed to the nearest 0.1 kg wearing school clothes without shoes during the first period of the school day on a calibrated scale (Model #PS6600, Befour, Saukville, WI). Standing height was measured with a portable stadiometer (Model #IP0955, Invicta Plastics Limited, Leicester, UK). Body mass index (BMI) was calculated as weight (kg)/height (m^2^). BMI percentile will be calculated using the CDC growth charts [27]. Waist circumference served as a surrogate for abdominal adiposity and was assessed using the procedures described by Lohman et al [28]. Three measurements were taken with the outcome recorded as the average of the closest 2 measures.

### Cardiovascular fitness

Cardiovascular fitness was assessed using the Progressive Aerobic Cardiovascular Endurance Run (PACER) used in FITNESSGRAM [29-31]. The PACER is a multistage fitness test based on a shuttle run that progresses in intensity as participants run 20-meters back and forth with a goal to run as long as possible. The reliability and validity of PACER cardiovascular fitness standards have been shown to be moderately high and acceptable [29]. A regression equation was used to estimate aerobic fitness (VO_2_ max) from equivalent 1-mile run time estimated from the total number of laps completed on the PACER [32].

### Attention-to-task

Attention-to-task will be assessed by trained research staff on the same random schedule for recording classroom physical activity, described in the process evaluation section below, using a modification of the procedures from Mahar et al [33]. During each assessment, approximately 5 students will be observed for 5 minute, 20 minutes before and 20 minutes after an A + PAAC activity or non-active lesson in control schools. Each child will be observed for 10 seconds, after which the appropriate behavior code will be circled. This procedure will be repeated over the 5 minute interval to result in a total of ~ 20 observations per child. Coding includes on-task, motor off-task, noise off-task, or passive/other off-task [34,35].

#### Daily physical activity: accelerometry

Children wore an ActiGraph GT1X portable accelerometer (ActiGraph LLC, Pensacola, FL) on a belt over the non-dominant hip for 4 consecutive days (including 1 weekend day). Accelerometer data was collected and summarized in 1-minute epochs with a minimum of 12 hours constituting a valid monitored day. ActiGraphs will be distributed to participants on Wednesdays and Fridays and returned to the school following completion of the 4-day monitoring period. The main outcome variable is the average ActiGraph counts/minute over the 4-day period. In addition, the average number of minutes/day over 4 days spent at various activity levels will be assessed applying the cut-points used in the National Health and Nutrition Examination Survey as described by Troiano et al [36]. Physical activity count/minute and intensity levels by time of day on both school and weekend days were assessed.

### Process evaluation

Ongoing process evaluation will be completed to monitor and document teacher training, the degree to which the intervention was implemented as designed (i.e., fidelity), and the level of engagement in A + PAAC lessons by both teachers and students over the 3-year intervention (i.e., dose). Quality and effectiveness of the teacher in-services will be assessed by self-report survey conducted 3 months following completion of the initial in-service, after teachers have experience implementing A + PAAC. To measure the extent of implementation of A + PAAC, classroom physical activity will be directly observed every other week in both intervention and control schools using the activity portion of System for Observing Fitness Instruction Time (SOFIT), which is a validated time-moment sampling technique [37]. The energy expenditure of in-class A + PAAC activities (METs) will be assessed via portable open-circuit, indirect calorimetry (COSMED K4b^2,^ Rome, Italy) in a volunteer sample of 40 students (20 boys, 20 girls). Harrell et al [38]. Has validated the K4b^2^ system in children aged 8–14 years by comparing VO_2_ obtained from the K4b^2^ with the results from a standard laboratory metabolic cart system during rest, walking, and running. Student characteristics that may affect intervention objectives will be assessed including age gender, race/ethnicity. At the end of each school year, administrators will complete a modified version of the School Health Questionnaire [39]. Also, principals/vice principals from both intervention and control schools will complete a 5-item survey regarding external or competing factors that may have influenced the objectives of the A + PAAC intervention, thus posing possible threats to internal validity [40], including changes in the number of minutes allocated to PE, adoption of any new course content intended to increase student physical activity.

## Results

### Analysis plan-primary aim: academic achievement

Changes (year 3 minus baseline) in academic achievement (WIAT-III comprehensive score) will be compared between students attending A + PAAC intervention and control schools using a modified two-sample *t*-test adjusted for the intra-class correlation (ICC) [41] with a type I error rate of 5%. Similar analysis will be completed for WIAT-III sub-scales and for each of the secondary endpoints (Kansas State Assessment, cognitive function, BMI, cardiovascular fitness, attention-to-task, daily physical activity). This longitudinal study will provide multiple assessments over time; therefore linear mixed models will be used to assess differences in between A + PAAC and control in the primary and secondary endpoints. A compound symmetric correlation structure for estimating the intraclass correlation coefficient (ICC) within a school is assumed.

### Analysis plan- secondary aim: mediation analysis

Mediation will be examined using the approach described by Baron and Kenny [42], and further extended by MacKinnon [43] and Brown [44]. We will determine the extent to which possible intervention effects on academic achievement (an a priori condition for mediation) might be explained by changes in cognitive function, BMI, cardiovascular fitness, attention-to-task, and daily physical activity.

### Exploratory analysis

In addition to mediation analysis, baseline characteristics (BMI, gender, age, race, ethnicity as well as baseline levels of the secondary outcomes [see mediation analysis section]) will be evaluated as predictors of change in academic achievement. Each of these variables will be included in a linear mixed model, assuming a compound symmetric correlation within each school. Stepwise regression techniques will be used to determine the “best” predictor model. “Best” will be determined by minimizing the Bayesian Information Criteria among all the potential predictor equations.

### Missing data

Predictive model-based multiple imputation will be used to impute missing data. Since the primary longitudinal models are a mixed linear regression, using a predictive model-based approach will allow for the use of more actual data to impute the missing data. The rate of missing data over time will be examined to determine if treatment, baseline weight, gender, age, and/or school are related to the missing data. If none of these factors is related to the missing data, it might be safe to assume data are missing completely at random.

### Sample size/power

The primary aim of this study is to compare change in academic achievement (comprehensive score) between the two treatments (A + PAAC vs. control). Power calculations were based on results from a secondary analysis of change in academic achievement from the completed PAAC study [10,11]. Since this is a cluster randomized design or an inherent ICC within the schools was assumed. The primary analysis will be conducted using a modified two-sample *t*-test [41] using a type I error rate of 5%. Table 3 describes the power for a range of ICCs, number of schools per treatment, and sample sizes within each school assuming an expected improvement in academic achievement of 5 points in the A + PAAC group, no change in the control group with a common standard deviation of 9 points, similar to results from the PAAC study [10].

**Table 3:** Statistical power

**Schools per treatment**	**Students per school**	**Intra-Class correlation**					
		**0**	**0.01**	**0.03**	**0.05**	**0.07**	**0.1**
6	20	0.99	0.98	0.93	0.87	0.8	0.71
6	30	0.99	0.99	0.97	0.92	0.86	0.76
7	20	0.99	0.99	0.96	0.91	0.86	0.78
7	30	0.99	0.99	0.99	0.95	0.9	0.82

An ICC in the range of 0.03 to 0.07 is anticipated for academic achievement, thus to ensure at least 85% power for this confirmatory study, 17 schools were randomized between the two treatment groups with 40 students being recruited per school. Based on the power analysis above, power will be sufficient with as few as 20 students per school and ICCs as high as 0.10. Given the limited preliminary data, exact power calculations are not possible for the secondary endpoints. However, assuming a type I error rate of 5%, secondary endpoints have the following power: 0.99, 0.99, 0.96, 0.91, 0.84 and 0.74, to detect an effect size of 0.5 standard deviations for ICCs equal to 0, 0.01, 0.03, 0.05, 0.07 and 0.10, respectively. Thus, the study will have sufficient power to detect any clinically meaningful difference on the secondary endpoints.

## Discussion

Cross-sectional studies suggest an association between physical activity and academic achievement in elementary school children [1,2]. However, results from the few available prospective studies on this topic are mixed [3-6]. The available studies have primarily evaluated the effect of increased PE which, in elementary school, is generally restricted to 30 minutes/day, 2 or 3 days/week, and provides limited time in MVPA. Thus, if physical activity is to be increased in elementary schools, venues other than PE need to be developed and evaluated.

Promoting increased physical activity using A + PAAC has a number of potential advantages. The elementary school setting provides an opportunity to increase the levels of physical activity in a large population of children. A + PAAC alters the classroom environment such that children are not required to make an overt choice to be more physically active, but do so as part of the academic curriculum. Therefore, the probability that children will actually engage in increased physical activity is likely higher than other approaches where participation is volitional (i.e., sports, after-school programs, etc.). The A + PAAC approach delivers academic lessons using physical activity, thus there is no reduction in academic instruction time; a desirable feature in the climate of No Child Left Behind of 2001 [45]. A + PAAC is minimal intervention that requires virtually no additional financial resources or time commitment to implement. Classroom teachers can be trained to deliver academic lessons through increased physical activity in the frequently used teacher in-service setting; a cost effective training model.

In summary, this project will evaluate the impact of physically active lessons in the elementary school classroom (A + PAAC) on academic achievement. If successful, the A + PAAC approach could be easily and inexpensively scaled and disseminated across elementary schools in the US using existing personnel and infrastructure to improve both educational quality and health.

## Abbreviations

A + PAAC: Academic achievement and physical activity across the curriculum; PE: Physical education; MVPA: Moderate to vigorous physical activity; WIAT: Weschler individual achievement test; BMI: Body mass index; MET: Metabolic equivalent of task; PACER: Progressive aerobic cardiovascular endurance run; SOFIT: System for observing fitness instruction time.

## Competing interests

The authors declare that they have no competing interests.

## Authors’ contributions

JED, RAW, CGG, DKS, JLG, DMH, CHH, BKS and JP conceived of the study, participated in the design and coordination and helped draft the manuscript. MSM participated in the design and statistical analyses. KL, SDH, JLB, JJH and MS participated in coordination and outcome assessments and helped draft the manuscript. All authors read and approved the final manuscript.

## Pre-publication history

The pre-publication history for this paper can be accessed here:

http://www.biomedcentral.com/1471-2458/13/307/prepub
